# Prevalence, Risk Factors and Association with Clinical Outcomes of Malnutrition and Sarcopenia in Inflammatory Bowel Disease: A Prospective Study

**DOI:** 10.3390/nu16233983

**Published:** 2024-11-21

**Authors:** Cristina Bezzio, Daniele Brinch, Davide Giuseppe Ribaldone, Maria Cappello, Natalie Ruzzon, Marta Vernero, Davide Scalvini, Laura Loy, Sofia Donghi, Stefania Ciminnisi, Gianpiero Manes, Alessandro Armuzzi, Simone Saibeni

**Affiliations:** 1 IBD Centre, IRCCS Humanitas Research Hospital, 20089 Rozzano, Italy; laura.loy@humanitas.it (L.L.); alessandro.armuzzi@hunimed.eu (A.A.); 2 Department of Biomedical Sciences, Humanitas University, Pieve Emanuele, 20072 Milan, Italy; 3 Gastroenterology Unit, Rho Hospital, ASST Rhodense, 20017 Rho, Italy; danielebrich@gmail.com (D.B.); natalie.ruzzon89@gmail.com (N.R.); sofia.donghi@studenti.unimi.it (S.D.); gmanes@asst-rhodense.it (G.M.); saibo@tiscali.it (S.S.); 4 Division of Gastroenterology, Department of Medical Sciences, Università di Torino, 10126 Turin, Italy; davide.ribaldone@unito.it (D.G.R.); martavernero@gmail.com (M.V.); 5 Gastroenterology and Hepatology Section, ProMiSe Department, University of Palermo, 90100 Palermo, Italy; marica.cappello61@gmail.com (M.C.); stefania.ciminnisi@gmail.com (S.C.); 6 Experimental Medicine, Department of Internal Medicine and Therapeutics, University of Pavia, 27100 Pavia, Italy; davidescalvini.med@gmail.com

**Keywords:** Crohn, ulcerative colitis, GLIM, MUST, SaskIBD

## Abstract

Introduction: The prevalences of malnutrition and sarcopenia in patients with IBD are not precisely known, and nutritional assessment is not standardized. We assessed the prevalence and risk factors of these conditions in outpatients and their impact on clinical outcomes. Methods: This prospective longitudinal study considered patients who had IBD for at least one year, were attending a tertiary IBD center, and were followed for the subsequent year. Results: In a sample of 158 consecutive patients (96 with Crohn’s disease and 62 with ulcerative colitis), the prevalence of malnutrition, according to GLIM criteria, was 13.3%. For identifying patients at risk of malnutrition, the Malnutrition Universal Screening Tool demonstrated better accuracy, (sensitivity 88.9 (65.3–98.6) and specificity 90.2 (83.8–93.4)) than the SaskIBD-NR questionnaire (sensitivity 69.3 (41.1–86.7) and specificity 60.9 (60.9–76.8)). The prevalence of sarcopenia was 34.2%. Considering clinical outcomes, sarcopenia at baseline was significantly associated with hospital admission within a year (*p* = 45.2% vs. 20.3%, 0.026). Conclusions: Malnutrition and sarcopenia were present in about one-third of IBD patients. Awareness should be raised among physicians caring for IBD patients about the need to evaluate patients’ nutritional statuses to help patients achieve a better quality of life.

## 1. Introduction

Inflammatory bowel diseases (IBD), including Crohn’s disease and ulcerative colitis, are chronic relapsing inflammatory conditions of the intestine. The etiology of these diseases is not fully understood, although according to the most accepted hypothesis, IBD results from altered immune responses to an imbalanced intestinal microbiome in genetically predisposed subjects [1,2,3]. The main symptoms of IBD are diarrhea, weight loss, abdominal pain (in Crohn’s disease), diarrhea, rectal bleeding, tenesmus, and urgency (in ulcerative colitis) [4,5].

According to the World Health Organization (WHO), malnutrition is defined as “deficiencies, excesses or imbalances in a person’s intake of energy and/or nutrients” and includes both undernutrition (i.e., poor growth, underweight status, micronutrient deficiencies) and overnutrition (i.e., overweight status, obesity, diet-related noncommunicable diseases, such as diabetes, heart disease) [6].

Malnutrition in IBD patients is commonly observed, yet its prevalence is far from established, with estimates ranging from 6% to 70% according to different definitions and in different clinical settings [7,8,9,10,11]. The prevalence is similar in Crohn’s disease and ulcerative colitis [12,13]. Although malnutrition is mainly associated with persistent clinical disease activity, it can also affect patients in clinical remission [8]. Malnutrition may arise from a combination of several factors [14]. Symptoms such as chronic abdominal pain and diarrhea often reduce food intake [15]. Moreover, many IBD patients are concerned about eating foods that could exacerbate their symptoms [16]; this fear can lead them to avoid specific food groups, leading to nutritional deficiencies. Persistent inflammation mediated by cytokines, such as tumor necrosis factor and interleukins 1 and 6, can increase catabolism and lead to anorexia, further exacerbating malnutrition [17]. Epithelial alterations and disease activity can induce inflammatory diarrhea, leading to direct nutrient loss [18]. Hospitalization (due to disease flares or complications) often involves fasting or dietary restrictions that contribute to nutritional deficits [19]. Surgical interventions, in particular ileal resection, are also associated with malnutrition, so early nutritional support in patients undergoing abdominal surgery is required [20]. In addition, we should keep in mind that also obesity is quite frequently observed in patients with IBD, with an estimated prevalence ranging between 15% and 40% that parallels the high frequencies observed in Western countries [21].

Malnutrition has negative consequences on an IBD patient’s life. It impairs growth and development in children [22], hinders the response to treatment [23], increases the risks of hospitalization and poor outcomes [24,25], and reduces the overall quality of life [26]. Also, obesity has been associated with negative clinical outcomes in IBD, such as a reduced response to treatment and increased rates of complications and postoperative infections [21]. Thus, the European Society for Clinical Nutrition and Metabolism (ESPEN) recommends regular nutritional follow-up for all IBD patients [27]. Nonetheless, a gold standard method for diagnosing malnutrition or malnutrition risk is lacking, especially in IBD [28]. A commonly used tool to assess malnutrition risk is the Malnutrition Universal Screening Tool (MUST), which considers body mass index (BMI), recent unintentional weight loss, and the disease’s effects on nutritional intake [29]. Other screening tools that are used include the Malnutrition Screening Tool (MST) for hospitalized patients, the Nutrition Risk Screening (NRS-2002) for critically ill patients, and the Mini Nutritional Assessment (MNA) for geriatric patients [30,31,32]. Recently, the Global Leadership Initiative on Malnutrition (GLIM) established consensus criteria for diagnosing malnutrition in different clinical settings [33]. GLIM criteria incorporate three phenotypic parameters, namely, low BMI, unintentional weight loss, and low fat-free mass index (FFMI), and two etiological parameters, namely, reduced food intake and inflammation. The Saskatchewan IBD–Nutrition Risk (SaskIBD-NR) tool, which is specific for IBD, considers factors such as disease activity, weight loss, dietary intake, and functional status [34].

Another emerging problem in the management of IBD patients is sarcopenia (i.e., loss of skeletal muscle mass or function). Sarcopenia is an emerging worldwide issue associated with various adverse health outcomes. It has been suggested that sarcopenia is associated with a high risk of poor survival rate, postoperative complications, and longer hospitalization in patients, as well as falls, fractures, metabolic disorders, cognitive impairment, and mortality in general populations. The prevalence of sarcopenia widely differs between studies, depending on the definitions used to define it; in the general population, the prevalence ranges from 5% to 22%, while it is higher in different patient groups (up to 83% in patients with esophageal cancer) [35]. The reported prevalence of sarcopenia in IBD patients ranges from 17% to 70%, and it is considered a risk factor for surgery, hospitalization, and postoperative complications [35,36,37]. Sarcopenia can be diagnosed using diagnostic imaging [38], but more practical and cost-effective methods involve the use of surrogate markers of muscle mass, such as handgrip strength and calf circumference. A peculiar form of malnutrition is represented by sarcopenic obesity; in IBD its prevalence has been estimated to be around 5% [39].

Given the negative impact of malnutrition and sarcopenia on patients with IBD and the yet inadequate information about their prevalences and associations, we designed a prospective multicenter longitudinal study to assess the prevalence of malnutrition and sarcopenia in a population of IBD outpatients, the risk factors for these associations, and the impact of malnutrition and sarcopenia on clinical outcomes after one year of follow-up. This study also investigated the accuracy of the MUST and SaskIBD-NT tools using GLIM criteria as the reference method.

## 2. Materials and Methods

This study was conducted at the tertiary IBD centers of Rho Hospital, A.O.U. Città della Salute e della Scienza of Turin and Policlinico of Palermo. Each center was able to provide local nutritional consultation.

Consecutive adult patients with established diagnoses of IBD of at least one year, according to ECCO guidelines [40], were enrolled between December 2022 and January 2023. Patients were excluded if they were pregnant or unable to understand and give informed consent. The included patients were followed for the subsequent year.

### 2.1. Research Ethics

The study design was approved by the Ethics Committee of Turin Hospital (Number of protocol 0138294, date 2 December 2022). Informed consent was obtained from all patients in this study.

### 2.2. Clinical Data Collection

For each patient, the following clinical data were collected at inclusion: age, sex, smoking, diagnosis (Crohn’s disease or ulcerative colitis), disease duration, previous and current therapy, previous abdominal surgery, ileal involvement > 30 cm (yes or no), steroid treatment (no, previous or current), and serum levels of albumin, ferritin, *C*-reactive protein (CRP), folate, vitamin B12, and 25-OH vitamin D. Extensive ileal involvement (>30) cm was considered.

For patients with Crohn’s disease, we collected information on disease location and behavior according to the Montreal criteria [41] and disease activity according to the Endoscopic Score for Crohn’s Disease (SES-CD) and the Harvey–Bradshaw index [42]. For patients with ulcerative colitis, we collected information on disease extent according to the Montreal criteria [41] and disease activity according to the partial Mayo (pMayo) score and endoscopic Mayo score [43].

At 54 weeks, the following clinical data were collected: hospital admission, need to change therapy (including need for steroids), and need for surgery.

### 2.3. Physical Examination and Nutritional Assessment

Weight and height were measured to calculate BMI. Waist and calf circumferences were measured using a plastic measuring tape while patients were seated with their feet on the floor and their knees and ankles at right angles. This measurement was taken at the location of the greatest circumference on a plane perpendicular to the long axis of the right calf.

Participants were instructed to squeeze a dynamometer as strongly as possible, and the instrument recorded the peak force in kilograms [44,45]. Maximum hand grip strength was obtained as the maximum value of three trials of the non-dominant hand, which were assessed with the elbow flexed at 90°. Low hand grip strength was defined as <27 kg for men and <16 kg for women.

To assess nutritional status, patients were asked if they had experienced unintentional weight loss in the previous 6 months or more. Body composition was assessed using a bio-impedance vector analysis (BIVA). Patients were interviewed to record their food- and nutrition-related history and determine their energy requirements.

### 2.4. Malnutrition and Sarcopenia Diagnoses

Malnutrition was diagnosed according to GLIM criteria [33] when patients met at least one phenotypical and one etiological criterion; phenotypical criteria are (i) BMI < 20 kg/m^2^ for patients < 70 years old and <22 kg/m^2^ if >70 years old; (ii) unintentional weight loss >5% within the past 6 months or >10% beyond 6 months; and (iii) a FFMI < 17 kg/m^2^ for men and <15 kg/m^2^ for women. Etiological criteria are (iv) reduced food intake or assimilation and (v) a state of inflammation.

The risk of malnutrition was predicted using two scores: MUST, which was categorized as low (0), medium (1), or high (>1) risk, and SaskIBD-NR, which was categorized as low (0–2), medium (3–4), or high (>4) [29,34]. Sarcopenia was diagnosed when patients had either low grip strength (<20 kg for women and <30 kg for men) or a calf size below the norm for their age [46,47].

### 2.5. Statistical Analyses

Data were analyzed using MedCalc statistical software version 20.104 (MedCalc Software, Ostend, Belgium). The distribution of continuous variables was tested for normality with the D’Agostino-Pearson test. Normally distributed variables were expressed as mean and standard deviation (SD), while skewed variables were reported as median and interquartile range (IQR).

Associations between groups and categorical variables were assessed using Fisher’s exact test. Univariate and multivariate analyses were conducted using stepwise logistic regression. Correlations between skewed variables were assessed with Spearman’s rank correlation. A receiver operating characteristic (ROC) curve analysis was used to evaluate the accuracy of the MUST and SaskIBD-NR scores, using a diagnosis of malnutrition according to the GLIM criteria as a reference. The Youden index was calculated to define the threshold that optimizes the sensitivity and specificity of the two screening tools. A *p*-value of <0.05 was considered statistically significant.

## 3. Results

The study sample consisted of 158 IBD patients (96 with Crohn’s disease and 62 with ulcerative colitis), with a mean age of 45.4 years (Table 1). There was a slight predominance of male patients (54.4%) and never-smokers (60.8%). The median disease duration was 8.1 years (IQR, 4–15 years). Of the 96 patients with Crohn’s disease, 56 (58.3%) had ileocolonic disease and 15 (15.6%) had perianal disease; according to the Harvey–Bradshaw index, 47 patients (49.0%) were in remission. Of the 62 patients with ulcerative colitis, 30 patients (48.4%) had left-sided colitis; according to the pMayo score, 30 patients (48.4%) were in remission.

### 3.1. Nutritional Status and Risk Factors for Malnutrition

Patients underwent a physical examination and nutritional assessment to determine their nutritional status (Table 2). According to BMI, 17 (10.8%) patients were considered undernourished and 13 (8.2%) were considered obese.

Unintentional weight loss of >10% was reported by nine patients (5.9%). According to the GLIM criteria, 21 patients had malnutrition, with an overall prevalence of 13.3%. This subgroup included 8 men and 13 women (*p* = 0.056), and there were 16 patients with Crohn’s disease and 5 with ulcerative colitis (*p* = 0.15).

In the univariate analysis, female sex, a Harvey–Bradshaw index of >4, and the current use of steroids were significantly associated with malnutrition (Table 3). Furthermore, the Harvey–Bradshaw index directly correlated with malnutrition, while waist circumference and albumin inversely correlated. There was no association with disease type, disease location, Crohn’s disease behavior, or extended ileal disease (ileal involvement > 30 cm). In a multivariate logistic regression, the associations were confirmed for waist circumference and albumin, with high values of these variables being inconsistent with malnutrition.

### 3.2. Assessment of Malnutrition Risk and Prediction of Malnutrition

**Figure 1:**
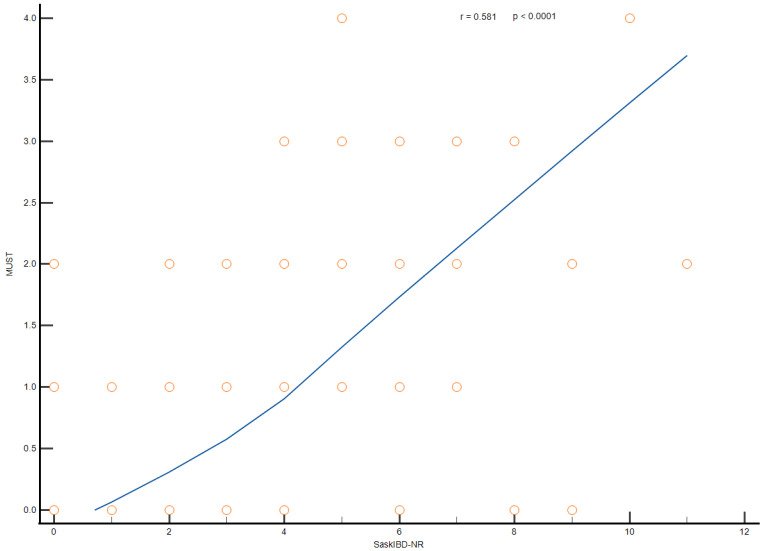
Correlation between MUST and Sask IBD in determining the risk of malnutrition. The circles in the graph represent integer numbers.

According to the MUST, 31 IBD patients (19.6%) were at a high risk of malnutrition. Similar results were obtained using the SaskIBD-NR tool (30, 18.9%) (Table 4). However, a higher percentage of patients were at moderate risk according to their SaskIBD-NR scores. MUST demonstrated good accuracy in predicting a diagnosis of malnutrition, with sensitivity and specificity of around 90%. SaskIBD-NR had low sensitivity (69.3%) but a high negative predictive value (94.2%). A moderate correlation was found between the MUST and SaskIBD-NR assessments (Spearman’s r = 0.581, *p* < 0.0001) (Figure 1).

**Figure 2:**
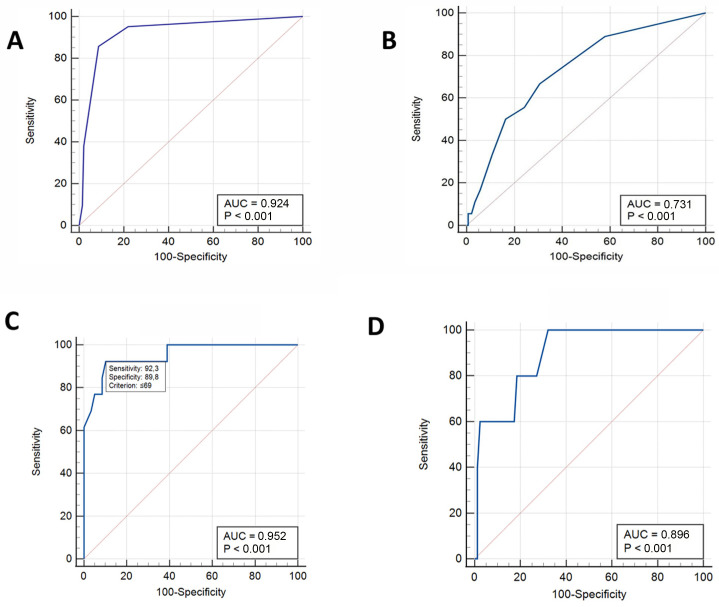
Receiver operating characteristic (ROC) curves to determine threshold values for predicting malnutrition. (**A**) MUST scores. (**B**) SaskIBD-NR scores. (**C**) Waist circumference for female patients. (**D**) Waist circumference for male patients.

A large ROC curve was observed for MUST (AUC = 0.924, *p* < 0.001), indicating that it has good accuracy in predicting malnutrition. SaskIBD-NR had lower accuracy in predicting malnutrition (AUC = 0.731, *p* < 0.001) (Figure 2A,B).

ROC curves were also constructed to determine threshold values of waist circumference for the risk of malnutrition. For female patients, a waist circumference of ≤69 cm identified malnutrition, with an AUC of 0.952, sensitivity of 92.3%, and specificity of 89.8%. For male patients, a waist circumference of ≤80 cm identified malnutrition, with an AUC of 0.896, sensitivity of 100%, and specificity of 68.0% (Figure 2C,D).

### 3.3. Sarcopenia Diagnosis and Association with Malnutrition

Overall, 55 out of 168 (34.8%) IBD patients were diagnosed with sarcopenia. The median age of these patients was 41 years (IQR 26–56). This subgroup included 27 women and 28 men, with a prevalence of 37.5% and 32.5%, respectively. Sarcopenia was present in 35 of 96 (36.4%) patients with Crohn’s disease; in 12 patients, the disease was located in the ileum, in 2 patients, it was located in the colon, in 19 patients, it was located in the ileum and the colon, and in 2 patients, it was located in the upper GI tract (both with concomitant colonic involvement). Sarcopenia was present in 20 of 72 (32.3%) patients with ulcerative colitis; in 20 patients, the disease was extended to the rectum, in 8 patients, it was extended to the left colon, and in 7 patients, it was extended to the entire colon.

In sarcopenic patients, the prevalence of malnutrition was 27.3% (15 of 55 cases). Furthermore, of the 21 IBD patients with malnutrition, 18 had sarcopenia. No sarcopenic patient was considered obese.

In a univariate analysis, sarcopenia was significantly associated with Crohn’s disease activity (OR = 1.67; 95% CI, 1.07–2.61; *p* < 0.031), extensive ileal disease (OR = 3.36; 95% CI, 1.40–8.04; *p* < 0.007), and previous surgery in Crohn’s disease (OR = 2.03; 95% CI, 1.99–4.17; *p* = 0.05). In a multivariate analysis, sarcopenia was significantly associated with extensive ileal disease (OR = 5.58; 95% CI, 1.03–30.20; *p* < 0.005), low serum albumin (OR = 0.11; 95% CI, 0.01–0.78; *p* < 0.03), and a small waist circumference (OR = 0.69; 95% CI, 0.54–0.89; *p* < 0.005).

### 3.4. Nutritional Consultation and Intervention; Association Between Sarcopenia–Malnutrition and Negative Clinical Outcomes

Patients diagnosed with malnutrition or sarcopenia at baseline were referred to a nutritionist, who introduced personalized nutritional support or education for each patient. In particular, undernourished patients with malnutrition were basically treated with oral nutritional supplements, leading to the normalization of nutritional parameters in all patients but two after one year. On the contrary, only two patients with baseline obesity showed a BMI of <30 Kg/m^2^ after one year. Patients with sarcopenia were treated in different manners, including isolated or combined high dietary protein intake (especially amino acids) and physical activity (both aerobic and resistance training). After one year, sarcopenia was still present in 37 of 55 patients.

**Figure 3:**
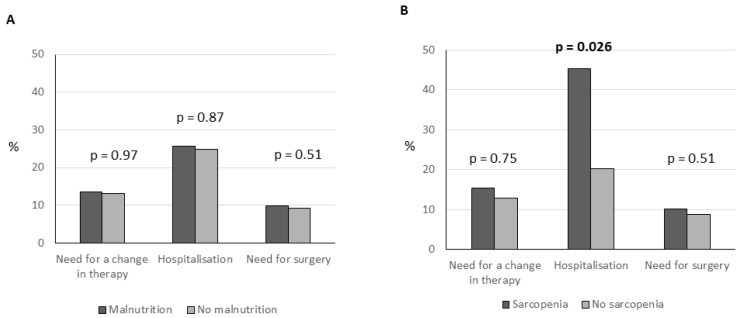
Association between presence of malnutrition (**A**) and sarcopenia (**B**) and negative outcomes within a year.

After 54 weeks of follow-up, the associations between baseline malnutrition and sarcopenia with clinical outcomes were assessed (Figure 3A,B). In univariate analyses, no significant associations were found between malnutrition and the need for a change in therapy (13.5% vs. 13.2%, *p* = 0.97), hospitalization (25.7% vs. 24.9% *p* = 0.87), or surgical interventions (9.8% vs. 8.3% *p* = 0.51).

Conversely, sarcopenia showed a significant association with hospital admission (45.2% vs. 20.3% *p* = 0.026). In contrast, no significant associations were found between sarcopenia and the need for a change in therapy (15.4% vs. 12.8%, *p* = 0.75) or the need for surgical intervention (10.1% vs. 8.7% *p* = 0.51)

Multivariate analyses reinforced these findings, demonstrating that malnutrition was not a significant predictor of any of the clinical outcomes examined (*p* > 0.05 for all outcomes). However, sarcopenia at baseline was associated with an increased risk of hospitalization within a year (adjusted odds ratio = 2.34, 95% CI: 1.10–4.98).

## 4. Discussion

In this cross-sectional study of 158 IBD patients, the prevalence of malnutrition was 13.3%. Factors associated with malnutrition were small waist circumference and low albumin levels. Specifically, waist circumference values of <69 cm in men and <80 cm in women were associated with malnutrition. For the assessment of malnutrition risk, the MUST was more accurate than the SaskIBD-NR questionnaire. However, there was a good correlation between these questionnaires in identifying malnutrition risk. The prevalence of sarcopenia in this population was 34.8%, with no significant difference between the subgroups with Crohn’s disease and ulcerative colitis or between males and females. Factors associated with sarcopenia in the multivariate analysis were the presence of extensive ileal disease, low serum albumin levels, and small waist circumference. The prevalence of sarcopenia was higher in IBD patients with malnutrition than in those without. After 54 weeks of follow-up, our results suggest that malnutrition in patients receiving adequate nutritional supplementation did not emerge as a significant predictor for adverse clinical outcomes. In contrast, sarcopenia was identified as the only factor associated with an increased risk of hospitalization. This finding is in agreement with several retrospective studies that indicated sarcopenia as a risk factor for several negative clinical outcomes in IBD, such as failure of therapy, increased risk of hospitalization, and increased risk of post-surgical complications [48]. However, we should keep in mind that these findings have not all been confirmed by other studies and that the quality of evidence of the association between sarcopenia and negative clinical outcomes is quite low, which is essentially due to the retrospective nature of most studies, the small sample sizes and the different definitions, and the impact of the assessment of sarcopenia on the association with IBD outcomes [49].

The reported prevalence of malnutrition in patients with IBD ranges from 6% to 70% [7,8,9,10,11,12]. In our cohort of IBD patients, the prevalence was 13.3%. This finding is consistent with a study that examined a similar IBD population [9] and lower than in a study in which the sample had more severe illness [10]. Because the criteria used to define malnutrition vary among studies, it is difficult to compare these results.

Regarding the nutritional tools examined in this study, the MUST confirmed its effectiveness as a screening tool for predicting malnutrition defined according to the GLIM criteria [50]. The SaskIBD-NR tool had an acceptable correlation with the MUST, but it has a low sensitivity, which is consistent with findings reported in another study [13,50,51].

According to a recent study [52], sarcopenia and malnutrition are often concomitant in IBD patients. The reported prevalence of sarcopenia in IBD patients ranges from 17% to 70%. This variability may be attributed to differences in study populations (e.g., hospitalized patients undergoing surgery vs. outpatients and patients in different geographical settings) [37,53,54,55]. However, the wide range of prevalence rates of sarcopenia mainly resides in the lack of a standardized definition of this condition and in the high heterogeneity in the modalities of assessing it [56]. Indeed, the presence of sarcopenia may be evaluated in several ways, for example, by assessing muscle strength, i.e., by means of hand grip or chair stand tests, or muscle mass, namely, appendicular skeletal muscle, total body skeletal mass, or cross-sectional area at the L3 level. In turn, muscle mass may be evaluated directly through DEXA scans, computed tomography, magnetic resonance imaging, and ultrasound and indirectly through bio-electrical impedance analyses. In addition, the normal cutoff for grip strength and muscle mass varies according to ethnicity and sex. In the present study, we observed a significant association between sarcopenia and extensive ileal Crohn’s disease, but we did not observe an association between sarcopenia and sex, age, or diagnosis. Indeed, the prevalence of sarcopenia was 36.4% in Crohn’s disease and 32.3% in ulcerative colitis). These findings are in agreement with previous reports that did not find differences in the prevalence of sarcopenia in patients with Crohn’s disease or ulcerative colitis, with reported prevalences of 35% and 32% [57], and 12% and 14% [58], respectively. Overall, the prevalence of sarcopenia in our study is in accordance with a previous study [54] and reflects the fact that the study population was composed of outpatients, about half of whom had active disease.

In our study, few patients were obese (8.2%), but we must remember that obesity is another form of malnutrition. The consideration of obesity, including sarcopenic obesity, is crucial, as it may alter patient management strategies in modern IBD care, mainly in Western countries. This is particularly important, as malnutrition is often associated with active disease phases, which means that stratifying patients by treatment type and disease activity level could provide a clearer understanding of the role of malnutrition in clinical outcomes.

The major strengths of this study are represented by its prospective nature, the homogeneous outpatient setting, and the use and comparison of several tools. At the same time, this study has some limitations. The main limitation is represented by the relatively small sample size, leading to the finding of non-homogeneous data (namely, the duration of disease, the rates of previous abdominal surgery, the ileal location of Crohn’s disease, and re-admissions) and resulting in potentially less reliable subgroup analyses. Other limitations are represented by the unorthodox (even if reliable) assessment of sarcopenia, the lack of power analysis, and the fact that the results may only be partially generalized to the entire population of IBD patients since they are representative of patients who have been referred to tertiary care centers.

This study underlines the importance of assessing the nutritional status of IBD patients; it has indeed been demonstrated that malnutrition has a negative impact on the quality of life of patients with IBD [37,52,58]. However, despite the recognized importance of nutritional status, in clinical practice, this assessment is not systematically performed by physicians caring for IBD patients [59]. In this direction, our study demonstrates that through the evaluation of easily assessable clinical parameters, such as waist and calf circumference, in addition to body weight and weight loss, and biochemical parameters, such as serum albumin, it is possible to assess the presence and the risk of malnutrition and sarcopenia. However, awareness should be raised among physicians caring for IBD patients about the need to evaluate patients’ nutritional status, mitigate the potential negative effects of malnutrition and sarcopenia, and help patients achieve a better quality of life.

## 5. Conclusions

In conclusion, our study confirms that malnutrition and sarcopenia are not rare in IBD patients. Nutritional status can initially be assessed from clinical variables that are easily measured, even by gastroenterologists. Since it is reported that malnutrition and sarcopenia are associated with worse clinical outcomes, the early identification of patients with malnutrition and sarcopenia and the implementation of specific interventions could improve the overall management of patients with IBD.

## Figures and Tables

**Table 1:** Clinical characteristics of 158 IBD patients (96 with Crohn’s disease and 62 with ulcerative colitis).

Characteristic			Value
Age, years, mean (SD)			45.4 (16.2)
Sex, n (%)			
	Male		86 (54.4)
	Female		72 (45.6)
Smoking habit, n (%)			
	Never		96 (60.8)
	Former		46 (29.1)
	Current		16 (10.1)
Disease duration, years, median (IQR)			8.1 (4–15)
Previous abdominal surgery, n (%)			45 (28.4)
Extended ileal disease, n (%)			36 (37.5)
Steroid treatment, n (%)			
	No		96 (60.8)
	Previous		46 (29.1)
	Current		16 (10.1)
Current biological therapy, n (%)			
	No		33 (20.9)
	Infliximab		56 (35.4)
	Adalimumab		24 (15.2)
	Vedolizumab		21 (13.3)
	Ustekinumab		24 (15.2)
Albumin, g/dL, median (IQR)			3.7 (3.3–4.2)
Ferritin, ng/mL, median (IQR)			48.5 (17–110)
*C*-reactive protein, mg/L, median (IQR)			2.1 (1.2–5.4)
Crohn’s disease patients (n = 96)			
	Disease location, n (%)		
		Ileal	28 (29.2)
		Colonic	8 (8.3)
		Ileocolonic	56 (58.3)
		Isolated upper disease	4 (4.2)
		Perianal disease	15 (15.6)
	Disease behavior, n (%)		
		Inflammatory	37 (38.5)
		Stricturing	36 (37.5)
		Penetrating	23 (24.0)
	Disease activity (Harvey-Bradshaw index), n (%)		
		Remission	47 (49.0)
		Mild	25 (26.0)
		Moderate	18 (18.7)
		Severe	6 (6.3)
Ulcerative colitis patients (n = 62)			
	Disease location, n (%)		
		Proctitis	10 (16.1)
		Left-sided colitis	30 (48.4)
		Extensive colitis	22 (35.5)
	Disease activity (pMayo score), n (%)		
		Remission	30 (48.4)
		Mild	18 (29.0)
		Moderate	13 (22.6)
		Severe	1 (0.02)

IQR, interquartile range.

**Table 2:** Physical and nutritional status of 158 IBD patients.

Characteristic		Value
BMI (kg/m^2^), median (IQR)		24.2 (20.9–28.3)
	<20 kg/m^2^ (for 152 patients < 70 years old), n (%)	15 (9.9)
	<22 kg/m^2^ (for 6 patients > 70 years old), n (%)	2 (33.3)
	>30 kg/m^2^, n (%)	13 (8.2)
Waist circumference, cm, median (IQR)		80 (72–94)
Calf circumference, cm, median (IQR)		34 (32–37)
Unintentional weight loss >10%, n (%)		9 (5.9)
Malnutrition according to GLIM criteria, n (%)		21 (13.3)
	Men	8 (9.3)
	Women	13 (18.1)
	Crohn’s disease	16 (16.7)
	Ulcerative colitis	5 (8.1)
Grip strength, kg, mean (SD)		31.96 (10.72)

BMI, body mass index; GLIM, Global Leadership Initiative on Malnutrition.

**Table 3:** Associations between clinical characteristics and malnutrition according to the GLIM criteria.

		Univariate Analysis		Multivariate Analysis	
		OR (95% CI)	*p* *	OR (95% CI)	*p*
Age		0.98 (0.95–1.01)	0.24		
Sex, male vs. female		0.28 (0.09–0.82)	0.021	5.58 (0.43–71.57)	0.186
Smoking habit, current vs. never		0.44 (0.05–3.70)	0.45		
CD vs. UC		1.83 (0.62–5.44)	0.27		
CD, colonic vs. ileocolonic		0.89 (1.02–7.91)	0.91		
CD behavior					
	Stricturing vs. inflammatory	1.65 (0.50–5.39)	0.40		
	Penetrating vs. inflammatory	1.71 (0.62–1.85)	0.47		
	Perianal disease	0.44 (0.05–3.70)	0.45		
Harvey-Bradshaw index		2.83 (1.49–5.38)	0.001	1.44 (0.43–4.82)	0.548
Extended ileal disease		1.48 (0.45–4.83)	0.51		
UC, proctitis vs. extensive colitis		1.26 (0.19–8.21)	0.80		
pMAYO score		3.01 (0.88–10.30)	0.07		
Steroid treatment, current vs. no		5.42 (1.41–20.8)	0.013	16.42 (0.22–18.21)	0.202
Previous abdominal surgery		1.70 (0.61–4.72)	0.30		
Waist circumference		0.72 (0.62–0.83)	<0.001	0.69 (0.54–0.89)	0.004
Disease duration		0.97 (0.92–1.03)	0.43		
Ferritin		1.01 (0.99–1.04)	0.98		
Albumin		0.09 (0.07–0.53)	0.007	0.03 (0.01–0.85)	0.040
*C*-reactive protein		0.98 (0.90–1.08)	0.79		

CD, Crohn’s disease; UC, ulcerative colitis. * Fisher’s exact test for categorical or categorized with cutoff data and Spearman’s for continuous data.

**Table 4:** Risk of malnutrition in 158 IBD patients and accuracy of MUST and SaskIBD-NR tools.

		MUST	SaskIBD-NR
Risk category			
	Low	107 (67.7)	75 (47.4)
	Medium	20 (12.6)	53 (33.5)
	High	31 (19.6)	30 (18.9)
Sensitivity (95% CI)		88.9 (65.3–98.6)	69.3 (41.1–86.7)
Specificity (95% CI)		90.2 (83.8–93.4)	60.9 (60.9–76.8)
Positive predictive value (95% CI)		53.3 (40.1–65.9)	21.8 (15.2–29.6)
Negative predictive value (95% CI)		94.8 (94.5–99.6)	94.2 (89.3–96.9)
Youden index		0.78	0.36

## Data Availability

The raw data supporting the conclusions of this article will be made available by the authors on request.
